# Editorial: Disease biomarker analysis based on optical biosensing

**DOI:** 10.3389/fchem.2023.1205533

**Published:** 2023-04-21

**Authors:** Tianshu Chen, Fanben Meng, Binwu Ying, Xiaoli Zhu

**Affiliations:** ^1^ Department of Clinical Laboratory Medicine, Shanghai Tenth People’s Hospital, School of Medicine, Tongji University, Shanghai, China; ^2^ Shanghai Children’s Medical Center, Department of Clinical Laboratory Medicine, School of Medicine, Shanghai Jiao Tong University, Shanghai, China; ^3^ Department of Mechanical and Materials Engineering, University of Nebraska-Lincoln, Lincoln, NE, United States; ^4^ Department of Laboratory Medicine, West China Hospital, Sichuan University, Chengdu, Sichuan, China

**Keywords:** disease biomarker, optical biosensing, molecular diagnostic, nano-biosensing, bioimaging

Disease biomarker analysis has become a crucial tool for diagnosing and evaluating disease prognosis, especially with the increasing understanding of diseases at the molecular level. Abnormalities in various biomarkers can indicate diseased states, and can be used to rapidly and specifically detect and quantify diseases using optical biosensing techniques (Gao et al., 2023). Optical biosensing techniques have several advantages over traditional methods including higher sensitivity, specificity, and faster analysis times (Plikusiene and Ramanaviciene, 2023). It also allows for non-invasive sample collection. With advancements in optical biosensing technology, many medical conditions including cancers, infectious diseases, and autoimmune disorders can be accurately diagnosed and efficiently treated (Singh et al., 2023; Tang et al., 2023). The combination of optical biosensing with emerging technologies such as material science, optics, and electronics has further accelerated its development in biomarker analysis (Qureshi et al., 2022). Interdisciplinary collaboration between experts in fields such as physics, chemistry, bioengineering, and medicine has helped pave the way for novel optical biosensing technologies as well as improving existing ones. Continued interdisciplinary collaboration is essential in advancing the field of disease biomarker analysis based on optical biosensing. This exciting area of research holds great potential for the future of personalized and precision medicine, and will likely lead to more effective disease diagnoses and treatments (Duo et al., 2023).

In order to highlight the most up-to-date research in this field, a Research Topic titled “Disease Biomarker Analysis Based on Optical Biosensing” was launched in Analytical Chemistry in Frontiers. The Research Topic consists of a total of 5 articles, which include 3 original research articles and 2 review articles. Through these publications, novel insights and significant discoveries are presented in the area of optical biosensing, contributing greatly to the advancement of this rapidly evolving field.

Bio-nanomicelles based on biomaterials such as nucleic acids, peptides, glycans, and lipids have rapidly developed in the field of bioanalysis (Yang et al., 2020). However, few bio-nanomicelles integrate DNA with peptides, which can provide unique advantages for biomedical applications. In light of this, Feng et al. designed a peptide-DNA hybrid bio-nanomicelle for the activity detection of caspase-3 (Zhang et al., 2022). They utilized the caspase-3 specific recognition and cleavage of peptide substrates to achieve high sensitivity and selectivity. Under optimized conditions, the detection of caspase-3 activity can be performed using their designed bio-nanomicelles with a detection limit of 0.72 nM. The proposed method was also successfully applied for the detection of caspase-3 in cell lysate samples after apoptosis-inducing. This work provides a valuable approach to integrate DNA with peptides for biomedical applications, and their bio-nanomicelles have the potential to be applied in a broad range of biological assays.

The detection of biomarkers, such as alpha-fetoprotein (AFP), plays a crucial role in the diagnosis and monitoring of various diseases (Dutta et al., 2021). Yan et al. have developed a highly sensitive and specific sensor for detecting AFP using electrochemiluminescence (ECL) and electrochemistry (EC) based on the gated transport of a bifunctional probe, Ru (phen)_3_Cl_2_, into the nanochannels of vertically ordered mesoporous silica films (VMSFs) (Chen et al., 2022). The VMSF surface possesses negatively charged properties and ultrasmall pore size, allowing for signal amplification and excellent sensitivity of the sensor. By covalently binding anti-AFP antibody to the external surface of VMSF through (3-glycidyloxypropyl) trimethoxysilane linkage, a specific sensing interface is created for the recognition of AFP. When AFP is present, the formed immunocomplex blocks the diffusion of Ru (phen)_3_Cl_2_ to the underlying electrode surface, resulting in a decreased ECL or EC response, allowing for a dual-mode detection of AFP with a low limit of detection and wide linear range. Furthermore, the inherent anti-fouling property of VMSF ensures satisfactory results in the analysis of human serum, demonstrating the potential of this strategy in clinical diagnosis. Overall, this study provides a promising approach for the detection of biomarkers using optical biosensing, which could have significant implications for disease diagnosis and prognosis evaluation.

Biosensors play a crucial role in disease diagnosis and screening, and the development of biosensing schemes that could simplify the design of biosensors is necessary to improve their efficiency. Li et al. have designed a peptide probe that can act as a “three-in-one” probe, allowing for target-binding, signal conversion, and amplification, which simplifies the design of biosensors (Jing et al., 2023). The probe design utilizes protein-triggered, conformation-driven, and Cu (II) facilitated side-chain di-tyrosine cyclization, enabling the use of target-probe recognition to induce cross-linking and self-cleavage of the probe, resulting in quantitative detection performance. The method does not require multi-step addition of enzymes, protein, and nanomaterial, which eliminates possible non-specific interactions and associated complexities. Preliminary testing of the probe in fractioned osteosarcoma clinical samples has shown acceptable coherence between signal readout and clinical diagnosis. The findings suggest that the proposed probe could be beneficial in the next development of tumor screening and prognosis sensors. The development of this peptide probe could revolutionize the design of biosensors by simplifying the process and improving their efficiency, leading to faster and more accurate disease diagnosis and prognosis evaluation.

DNA-modified AuNPs (DNA-AuNPs) have been extensively studied in the past decade and exhibit exciting features and bright prospects in many fields. Jiao et al. reviewed the various approaches for immobilizing DNA strands on the surface of AuNPs (Ma et al., 2022). Representative studies based on DNA-AuNPs for biomedical applications are also discussed. Finally, they discussed the challenges and future directions. This study highlights the potential of DNA-AuNPs for broad biomedical applications, providing researchers with new insights into the development and optimization of such systems. In another review article, Jiang et al. discussed the application of photoacoustic imaging in oncology, which enables visualization, characterization, and measurement of biological processes at the molecular and cellular level (Zheng et al., 2022). This review article discussed the three categories of photoacoustic imaging, namely, photoacoustic microscopy, photoacoustic tomography, and photoacoustic endoscopy, and highlights the advantages of this technique over traditional imaging methods in terms of improved imaging depth and resolution. The authors also reviewed recent advancements in targeted photoacoustic contrast agents and their application in tumor molecular imaging research. The article concluded with a discussion of the potential of photoacoustic imaging technology to facilitate the development of targeted therapies for cancer by providing precise diagnosis and treatment guidance.

Overall, this Research Topic have provided a variety of novel optical biosensing technologies for disease biomarker analysis with high sensitivity, specificity, and affordability. In addition, this Research Topic have discussed recent advances in disease biomarker analysis based on optical biosensing, summarizes the current state of the field, and highlights the challenges and opportunities that lie ahead.
